# Supplementary material for: Calibration of multi-layered probes with low/high magnetic moments
Source: Sci Rep. 2017 Aug 3;7:7224. doi: 10.1038/s41598-017-07327-0 (PMC5543078; doi:10.1038/s41598-017-07327-0)
Supplement: Supplementary file 1 — Supplementary Information [file 41598_2017_7327_MOESM1_ESM.pdf]

Calibration of multi-layered probes with low/high magnetic moments (Supplementary Information)

Vishal Panchal[1], Héctor Corte-León[1,2], Boris Gribkov[1,3], Luis Alfredo Rodriguez[4,5], Etienne Snoeck[4], Alessandra Manzin[6], Enrico Simonetto[6,7], Silvia Vock[8], Volker Neu[8], and Olga Kazakova[1*]

[1]National Physical Laboratory, Teddington, Hampton Road, TW11 0LW, United Kingdom

[2]Royal Holloway, University of London, Egham Hill, Egham TW20 0EX, United Kingdom

[3]Institute for Physics of Microstructures RAS, Nizhny Novgorod, 603950, Russia

[4]CEMES-CNRS, 29 Rue Jeanne Marvig, B.P. 94347, F-31055, Toulouse, France

[5]Department of Physics, Universidad del Valle, A. A. 25360, Cali, Colombia

[6]Istituto Nazionale di Ricerca Metrologica, I-10135, Torino, Italy

[7]Politecnico di Torino, I-10129, Torino, Italy

[8]Leibniz Institute for Solid State and Materials Research, D-01069 Dresden, Germany

*olga.kazakova@npl.co.uk

Spatial resolution of thin ML-MFM probe in FM and A-FM states

In order to establish the spatial resolution of the thin multi-layered (ML) magnetic force microscopy (MFM) probe (15/10/15 nm of Co/Si/Co layers), we imaged a hard disk drive (HDD) sample with the bit size of 30 nm. Figures S1a-S1d show MFM phase images in ±ferromagnetic (FM) and ±anti-ferromagnetic (A-FM) states of the probe, and Figs. S1e and S1f depict the line profiles extracted along the exact same region on the sample (as indicated by black, green, red and blue line profiles in Figs. S1a-S1d). As evident from these results, imaging with the FM state of the probe results in MFM phase changes that are up to four times larger than with the A-FM state. It should be noted that the difference in the MFM phase signal between the FM and A-FM states of the probe for floppy disk (two times large phase changes, Fig. 1 in the main text) and HDD sample (four times larger phase changes) is likely to be attributed to differences in their material properties and also their magnetisation direction (*i.e.*, floppy disk: parallel recording and HDD: perpendicular recording). The lateral spatial resolution of the thin ML-MFM probe in ±FM and ±A-FM states was determined from MFM phase line

profiles by taking the distance between points of well-defined relative intensity (20%–80%) as defined by the Edge Spread Function in Section 4.1.3 of Standards on Lateral Resolution (PD ISO/TR 19319:2013: *Surface chemical analysis – Fundamental approaches to determination of lateral resolution and sharpness in beam-based methods*). The results are summarised in Table S1. The average lateral spatial resolutions of thin ML-MFM probe in ±FM and ±A-FM states are 21.4±4.1 nm and 12.6±2.2 nm, respectively. Thus, A-FM state can resolve 1.7 times smaller features as compared to the FM state.

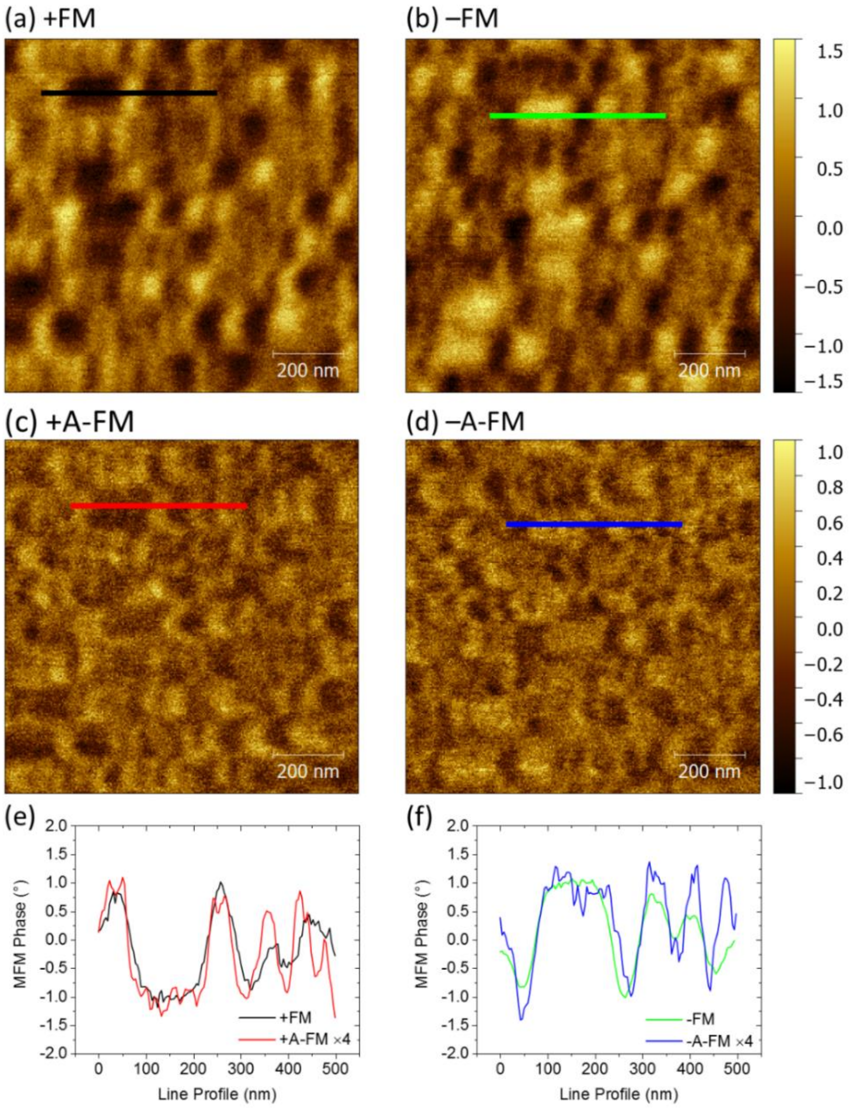

Figure S1. MFM phase images of a reference hard disk drive sample obtained with a thin ML-MFM probe (15/10/15 nm of Co/Si/Co layers) in four different configurations. (a) +FM, (b) −FM, (c) +A-FM and (d) −A-FM. Line profiles for (e) +FM and +A-FM and (f) −FM and −A-FM were obtained along the lines of the corresponding colour in (a)-(d), respectively. Note: the MFM phase signal for the ±A-FM state in (e) and (f), respectively, is multiplied by factor of four for easier visualisation of the data.

Table S1. Lateral spatial resolution of the thin ML-MFM probe in ±FM and ±A-FM states obtained from 9 different locations of the MFM phase images from Figs. S1a-S1d. The distance between points of well-defined relative intensity of 20 % and 80 % was used. Average lateral spatial resolution of thin ML-MFM probe in ±FM and ±A-FM states is 21.4±4.1 nm and 12.6±2.2 nm, respectively.

	+FM (nm)	−FM (nm)	+A-FM (nm)	−A-FM (nm)
	21.1	24.3	14.7	10.1
	21.9	24.7	13.3	13.9
	23.4	23.3	14.7	10.6
	19.6	20.9	11.0	13.4
	14.5	16.2	9.8	11.0
	19.6	14.7	15.9	14.0
	27.4	26.8	16.6	11.1
	26.3	24.8	12.5	12.3
	17.0	18.4	12.7	8.7
Average	21.4		12.6	
Standard Deviation	4.1		2.2	

RSTTF of thick ML-MFM probe

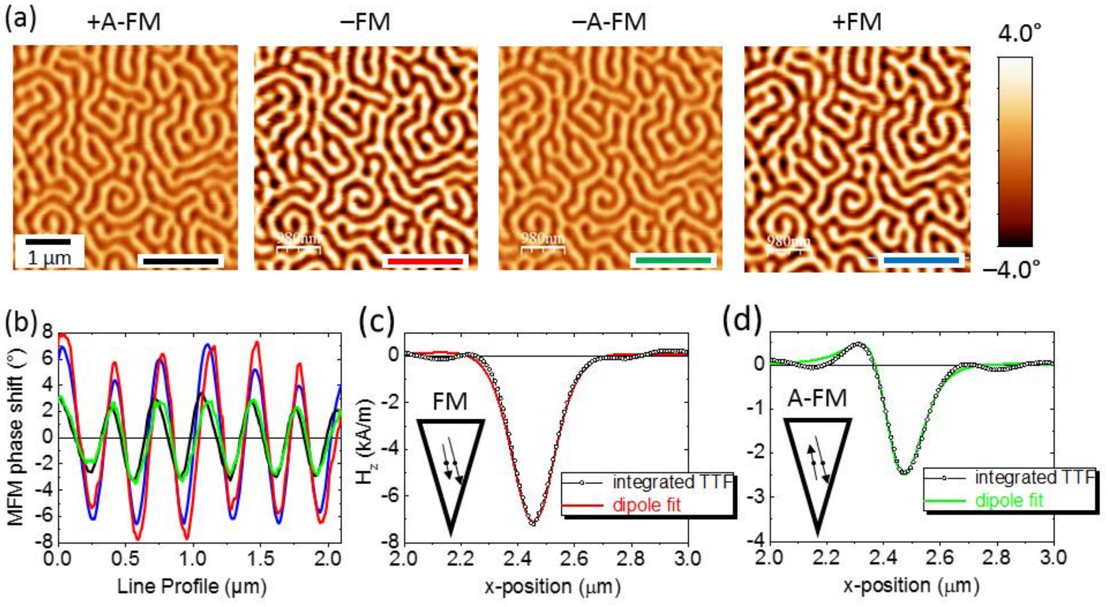

Figure S2. (a) MFM phase shift measurements on the Co/Pt reference sample using the thick (30/10/30 nm Co/Si/Co) ML-MFM probe magnetised in the (left to right) +A-FM, –FM, –A-FM and +FM states, respectively. (b) MFM profiles obtained along the lines of the corresponding colour in (a). Stray field profile (integrated RSTTF) of the thick ML-MFM probe in the (c) FM and (d) A-FM states, 55 nm below the tip apex.

Electron holography images of the thin ML-MFM probe

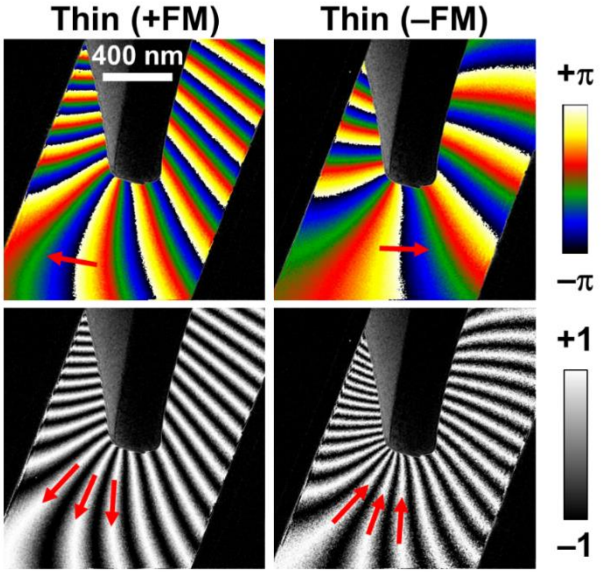

Figure S3. EH images taken near the apex of the thin ML-MFM probe. Colour images correspond to the magnetic phase shift, while black and white images represent the configuration of the magnetic flux due to the stray field. Arrows in the phase shift and magnetic flux images indicate the direction of the phase shift gradient and magnetic flux, respectively.

Experimental and simulated magnetic SGM maps of local Hall voltage

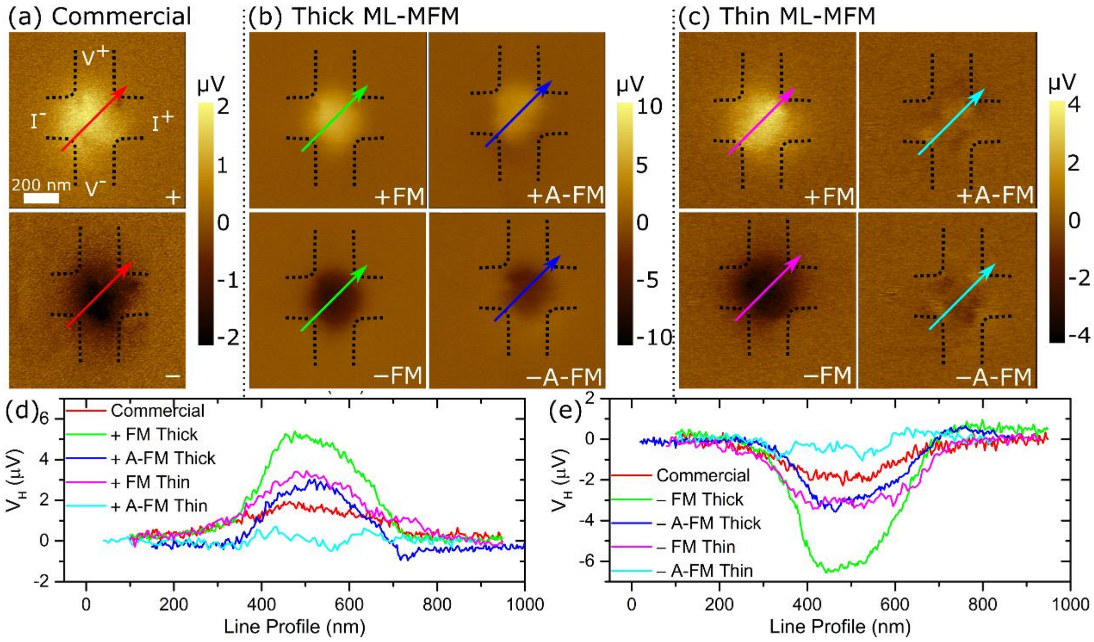

Figure S4. Same magnetic SGM images for (a) commercial Nanosensors MFM probe, (b) ML-MFM probe with 30/10/30 nm (thick) and (c) 15/10/15 nm (thin) Co/Si/Co layers in the FM state (↓↓) and A-FM state (↓↑), as shown in Fig. 6 in the main text. Top/bottom row of images in (a), (b) and (c) are for probes magnetized up/down, respectively. Black dashed lines depict the Hall cross borders. The electrical connections shown in (a) are the same for all other images. (d) and (e) are line profiles of the Hall voltage across the lines indicated in (a)-(c) for the probes magnetised in + and – direction, respectively.

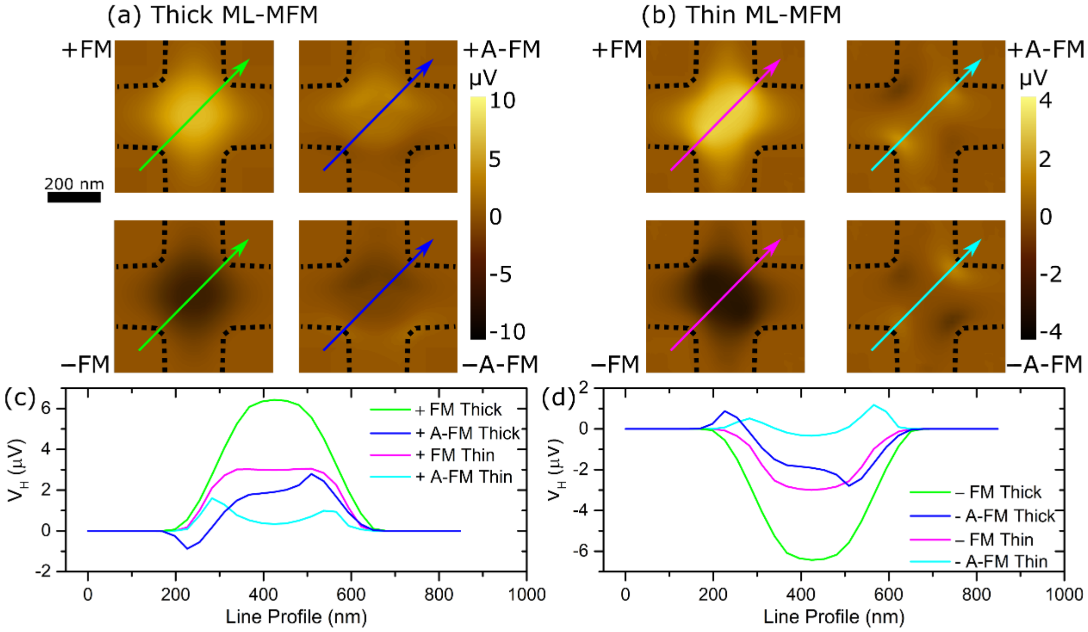

Figure S5. Same simulated magnetic SGM images for (a) ML-MFM probe with 30/10/30 nm (thick) and (c) 15/10/15 nm (thin) Co/Si/Co layers in the FM state (↓↓) and A-FM state (↓↑), as shown in Fig. 7 in the main text. Top/bottom row of images in (a) and (b) are for probes magnetised +/–, respectively. (c) and (d) are line profiles of the Hall voltage across the lines indicated in (a) and (b) for the probes magnetised in + and – direction, respectively.
